# Differential Mitochondrial Redox Responses to the Inhibition of NAD^+^ Salvage Pathway of Triple Negative Breast Cancer Cells

**DOI:** 10.3390/cancers17010007

**Published:** 2024-12-24

**Authors:** Jack Kollmar, Junmei Xu, Diego Gonzalves, Joseph A. Baur, Lin Z. Li, Julia Tchou, He N. Xu

**Affiliations:** 1 Department of Surgery, Perelman School of Medicine, University of Pennsylvania, Philadelphia, PA 19104, USA; jack.kollmar@pennmedicine.upenn.edu (J.K.); dgonzalv@sas.upenn.edu (D.G.); julia.tchou@pennmedicine.upenn.edu (J.T.); 2 Britton Chance Laboratory of Redox Imaging, Department of Radiology, Perelman School of Medicine, University of Pennsylvania, Philadelphia, PA 19104, USA; junmei.xu@pennmedicine.upenn.edu (J.X.); linli@pennmedicine.upenn.edu (L.Z.L.); 3 Department of Physiology and Institute for Diabetes, Obesity, and Metabolism, Perelman School of Medicine, University of Pennsylvania, Philadelphia, PA 19104, USA; baur@pennmedicine.upenn.edu

**Keywords:** mitochondrial redox state, optical redox ratio, FAD-containing flavoproteins, NAD^+^ biosynthesis, Nampt, label-free optical redox/metabolic imaging, FK866, GMX1778, TNBC

## Abstract

**Background/Objectives**: Cancer cells rely on metabolic reprogramming that is supported by altered mitochondrial redox status and an increased demand for NAD^+^. Over expression of Nampt, the rate-limiting enzyme of the NAD^+^ biosynthesis salvage pathway, is common in breast cancer cells, and more so in triple negative breast cancer (TNBC) cells. Targeting the salvage pathway has been pursued for cancer therapy. However, TNBC cells have heterogeneous responses to Nampt inhibition, which contributes to the diverse outcomes. There is a lack of imaging biomarkers to differentiate among TNBC cells under metabolic stress and identify which are responsive. We aimed to characterize and differentiate among a panel of TNBC cell lines under NAD-deficient stress and identify which subtypes are more dependent on the NAD salvage pathway. **Methods**: Optical redox imaging (ORI), a label-free live cell imaging microscopy technique was utilized to acquire intrinsic fluorescence intensities of NADH and FAD-containing flavoproteins (Fp) thus the mitochondrial redox ratio Fp/(NADH + Fp) in a panel of TNBC cell lines. Various fluorescence probes were then added to the cultures to image the mitochondrial ROS, mitochondrial membrane potential, mitochondrial mass, and cell number. **Results**: Various TNBC subtypes are sensitive to Nampt inhibition in a dose- and time-dependent manner, they have differential mitochondrial redox responses; furthermore, the mitochondrial redox indices linearly correlated with mitochondrial ROS induced by various doses of a Nampt inhibitor. Moreover, the changes in the redox indices correlated with growth inhibition. Additionally, the redox state was found fully recovered after removing the Nampt inhibitor. **Conclusions**: This study supports the utility of ORI in rapid metabolic phenotyping of TNBC cells under NAD-deficient stress to identify responsive cells and biomarkers of treatment response, facilitating combination therapy strategies.

## Simple Summary

Triple negative breast cancer (TNBC) is a heterogeneous disease with diverse treatment responses and poorer outcomes compared to receptor-positive breast cancer. We aimed to characterize the mitochondrial redox responses of several subtypes of TNBC cells under NAD-deficient stress using an optical redox imaging technique. We observed significant but differential alterations in the mitochondrial redox status. Our findings may aid in identifying responsive cells and biomarkers of treatment response, enabling combination therapy strategies through rapid metabolic phenotyping of TNBC cells under NAD-deficient stress caused by pharmacological inhibition of the NAD biosynthesis salvage pathway.

## 1. Introduction

Triple negative breast cancer (TNBC) is a subtype of breast cancer that lacks expression of estrogen receptors (ER), progesterone receptors (PR), and human epidermal growth factor receptor 2 (HER2). TNBC is a heterogeneous disease and shows diverse treatment responses and poorer outcomes compared to receptor-positive breast cancer. TNBC cells heavily depend on aerobic glycolysis, highlighting the potential of identifying early metabolic endpoints to predict long-term clinical outcomes. During treatment, the metabolic landscape of TNBC cells exhibit unstable spatial and temporal dynamics, with surviving populations adopting diverse metabolic states [1]. Research suggests that profiling the metabolism of TNBC could shed light on these variations and offer potential targets for intervention [2,3].

Through comprehensive gene expression analysis, four to six distinct molecular TNBC subtypes have been identified: basal-like (BL1 and BL2), mesenchymal and mesenchymal-like (M and ML), luminal androgen receptor (LAR), and immunomodulatory (IM) subtypes [4,5]. These subtypes with unique expression signatures and ontologies are likely to display differential metabolic phenotypes and adaptivity that contribute to the heterogenous treatment response and prognosis. Thus, better understanding the unique and common metabolic features of these TNBC subtypes is crucial for the identification of novel treatment strategies for TNBC patients. Furthermore, metabolic phenotyping may assist the development of non-invasive imaging techniques to enhance current histopathological prognostication [6].

Nicotinamide adenine dinucleotide (NAD, including the oxidized form NAD^+^ and the reduced form NADH) is indispensable for cellular energy and redox metabolism. It serves as a critical regulator of cellular function, linking metabolism to energy needs, signaling, and redox homeostasis [7,8]. NADH as a central electron donor in the mitochondrial electron transport chain (ETC) transfers high-energy electrons derived from metabolic pathways such as glycolysis, the citric acid cycle, and β-oxidation to the electron transport chain (ETC) to facilitate ATP generation through oxidative phosphorylation. NAD as a critical redox cofactor in redox reactions alternates between NAD^+^ and NADH. During glycolysis where glucose is converted into pyruvate, NAD^+^; is reduced to NADH, which in turn is oxidized to NAD^+^; in the last step of glycolysis when pyruvate is converted to lactate catalyzed by lactate dehydrogenase A (LDHA). The citric acid cycle produces high-energy electron carriers: NADH and reduced flavin adenine dinucleotide FADH_2_, which feed into the ETC for ATP production. The NAD^+^;/NADH redox ratio plays a crucial role in regulating ATP generation by influencing multiple steps in cellular metabolism and energy production. This ratio reflects the balance between NAD^+^; and NADH and serves as a key indicator of the cellular metabolic state. Besides its role as an essential redox co-enzyme, NAD^+^ also serves as a co-substrate for several classes of signaling enzymes including the poly (ADP-ribose) polymerases (PARPs), CD38, and sirtuins [9,10,11,12].

Optical redox imaging (ORI) or optical metabolic imaging (OMI) is a technique that collects the intrinsic fluorescence of protein-bound NADH and oxidized FAD-containing flavoproteins (Fp) and provides a measure of the mitochondrial redox state and cellular metabolic state represented by the mitochondrial redox ratio Fp/(NADH + Fp) (also referred to as the optical redox ratio) [13,14,15,16,17]. Imaging the fluorescence intensity or lifetime of NADH and FAD using single-photon or two-photon excitation provides biomarkers for assessing cellular metabolism, treatment response, and the pathological state of breast cancer. For example, differentiation among breast cancer cells with different receptor status by the basal mitochondrial redox ratio [18], identification of glycolytic levels, subtypes, and early-treatment response in breast cancer cells [19], detection of the effects of HER2 overexpression on breast cancer cell metabolism and treatment response [20], identification of tumor cell-cycle status [21] and singling out drug-responsive from drug-resistant cells [22]. Imaging of NADH and FAD has also been developed for intravital imaging to monitor immunotherapeutic response in TNBC [23]. However, imaging biomarkers to differentiate among metabolic phenotypes of TNBC subtypes under various metabolic stress are less reported. We previously showed that the basal mitochondrial redox ratio (Fp/(NADH + Fp)) differs among four TNBC cell lines under normal growth conditions and the more oxidized redox state corresponds to a higher invasive potential of the TNBC cells [24], suggesting a potential link between the mitochondrial redox state and TNBC metastatic potential.

**Figure 1:**
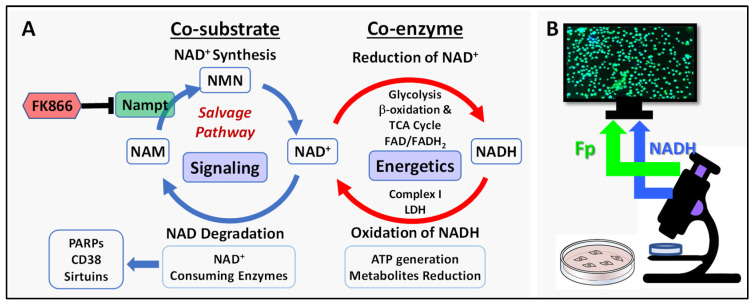
The critical role of NAD as a co-enzyme and co-substrate (**A**) and experimental schematic (**B**), where NAM stands for nicotinamide, NMN for nicotinamide mononucleotide, TCA for tricarboxylic acid cycle, LDH for lactate dehydrogenase.

TNBC cells have profound metabolic reprograming characterized by decreased mitochondrial respiration, increased glycolysis [25,26], and an increased demand for NAD metabolism [3]. The NAD salvage pathway (Figure 1A) is the primary mechanism that produces NAD^+^ and nicotinamide phosphoribosyltransferase (Nampt) is the rate-limiting enzyme of this pathway. Research into cancer therapies targeting NAD metabolism has been an area of intense interest [27,28]. Nampt is often overexpressed in breast tumors and more so in triple negative breast cancer (TNBC) cells, and high Nampt expression is associated with poorer survival [3,29,30,31]. NAD^+^ biosynthesis metabolism was also found to predict prognosis and indicates the state and composition of tumor immune microenvironment for breast cancer [32]. Inhibiting Nampt (thus inhibiting NAD^+^ regeneration) results in reduced serine biosynthesis from glucose, ATP depletion, suppressed proliferation, and ultimately, cell death [3,33,34,35,36]. However, TNBC cells are heterogeneous and can exhibit variability and adaptive potential under various metabolic stress.

Metabolic phenotyping to identify the responsive TNBC subtypes under metabolic stress for combination therapy and potential treatment response biomarkers facilitates the development of new therapeutic strategies. Previously, we found that inhibiting Nampt significantly decreased NADH and increased Fp and the redox ratio (Fp/(NADH + Fp)) in one TNBC cell line, which correlated with retarded growth, increased reactive oxygen species (ROS), and enhanced therapeutic effect of paclitaxel [35]. These results indicate the potential of ORI as a rapid metabolic phenotyping tool. In the present study, we aimed to test the capacity of ORI to characterize and differentiate among a panel of TNBC cell lines under NAD-deficient stress induced by pharmacological Nampt inhibitors FX11 or GMX1778. We also explored the correlations between alterations of the mitochondrial redox indices and other mitochondrial properties and cell growth inhibition.

## 2. Materials and Methods

### 2.1. Cell Preparation and Treatment

Human TNBC lines MDA-MB-468 (BL1), HCC1806 (BL2), MDA-MB-436 (MSL), MDA-MB-231 (MSL), and MDA-MB-453 (LAR) were purchased from ATCC (Manassas, VA, USA). MDA-MB-468 was cultured in Leibovitz’s L-15 medium and all the other human TNBC cell lines were cultured in RPMI 1640 (Gibco Cat # 11875085, Thermo Fisher Scientific, Waltham, MA, USA). All the growth media were supplemented with 10% FBS (MilliporeSigma, St. Louis, MO, USA). Murine breast tumor line E0771 and AT-3 were purchased from CH3 Biosystems (Amherst, New York, NY, USA) and Sigma-Aldrich (MilliporeSigma, St. Louis, MO, USA), respectively. E0771 cells were cultured in R10 medium (RPMI1640 GlutaMAX supplemented with 10% HI-FBS, 2 mM pyruvate, 1 × MEM non-essential amino acids, 10 mM HEPES). AT-3 cells were cultured in DMEM (Corning Cat. # 10-013) base medium supplemented with 10% HI-FPS, 15 mM HEPES, 2 mM MEM non-essential amino acids, and 0.1 mM beta-mercapto-ethanol. All cultures were maintained in 37 °C and 5% CO_2_ in T-flasks, except that MDA-MB-468 cells were cultured without CO_2_. For imaging, an appropriate number of cells were seeded in glass-bottom dishes (Cellvis, Mountain View, CA, USA). Thirty minutes before imaging, cells in the glass-bottom dishes were rinsed twice with DPBS and maintained in 1 mL Live Cell Imaging Solution (Invitrogen™, Carlsbad, CA, USA, Cat.# A59688DJ) supplemented with 11 mM glucose and 2 mM L-glutamine (LCIS^+^) for imaging.

FK866 and GMX1778 (Cayman Chemical, Ann Arbor, MI, USA) powder forms were dissolved in DMSO at their maximum solubilities, aliquoted, and stored in −20 °C until use.

For total NAD quantification, cells were grown and treated in tissue culture-treated 6-well plates. After the allotted treatment time, adhered cells were mechanically suspended using a cell scraper and removed from the wells. The cell suspensions were then processed using the fluorometric NAD^+^/NADH Assay Kit MAK460 (MilliporeSigma, St. Louis, MO, USA) as per manufacturer instructions to determine NAD^+^ and NADH concentration. A total of 150 thousand cells were used per sample, which was previously confirmed via titration to fit the linear regime of the assay.

### 2.2. Imaging, Data Processing, and Statistical Analysis

ORI acquisition and data processing have been described in detail in our previous works [35,37,38]. Briefly, redox images from 3 to 5 randomly chosen field of views (FOVs) were acquired using a wide field fluorescence microscope (Zeiss, Observer 7, White Plains, NY, USA) with the temperature chamber set at 37 °C. Transmitting light was first used to locate and focus on regions of interest to avoid photo-bleaching. The vignette effect was corrected on the fly. The imaging settings are listed in Table 1.

For the redox titration experiment (results are found in the Appendix A), mitochondrial metabolic perturbation drugs trifluoromethoxy carbonylcyanide phenylhydrazone (FCCP, 0.5 µM) and a mixture of rotenone (1 µM) and antimycin A (1.25 µg/mL) (ROT/AA) were sequentially added. Images were taken approximately ~2 min post addition of each drug. All the drugs were purchased from MilliporeSigma (St. Louis, MO, USA).

For detecting mitochondrial superoxide, MitoSOX Red (Thermo Fisher Scientific, Waltham, MA, USA) was added to the dishes after ORI with a final concentration of 2 µM and incubated at 37 °C for 10 min in the dark then rinsed once with PBS followed by imaging in 1 mL PBS.

For detecting mitochondrial membrane potential, TMRE (Thermo Fisher Scientific, Waltham, MA, USA) was added to the dishes after ORI with a final concentration of 200 nM and incubated at 37 °C for 20 min in the dark then rinsed once with PBS followed by imaging 1 mL PBS.

For detecting mitochondrial mass, MitoView Green (Biotium, Inc., Fremont, CA, USA) was added to the dishes after ORI with a final concentration of 200 nM and incubated at 37 °C for 20 min in the dark then rinsed once with PBS followed by imaging in 1 mL PBS.

To determine the cell number in the dish, Hoechst 33342 was added to the dish at a final concentration of 300 nM after ORI to label nuclei and whole dishes were imaged and counted.

The raw images (14-bit) were processed using a customized MATLAB^®^ (Version 2019a, The MathWorks, Inc., Natick, MA, USA) program to first remove the background by drawing a region of interest in a cell-free area and then threshold the pixel intensities on a signal-to-noise ratio (SNR) of 7.5 to obtain the net values of NADH and Fp as well as the pixel-by-pixel based redox ratio, whereas a SNR of 5 was applied for obtaining the MitoSOX signal; then, the mean values of NADH, Fp, redox ratio, and MitoSOX signal were computed for each FOV. The mean values of the imaging indices from each FOV were averaged to obtain dish means, which were then averaged across all dishes to calculate the group means. Biological control dishes were included in all imaging sessions, and data were normalized to the respective control dish values and expressed as fold change.

The group means were compared with ordinary one-way ANOVA with Bonferroni’s multiple comparisons test using GraphPad PRISM (v.10). *p* < 0.05 is considered statistically significant.

## 3. Results

### 3.1. Differential Mitochondrial Redox Responses to Nampt Inhibition

The mitochondrial redox state is an important parameter of mitochondrial function and cell metabolism. NAD, as a co-enzyme and co-substrate, plays a crucial role both in redox reactions and cell signaling, linking redox-dependent reactions critical for metabolism and bioenergetics (Figure 1A). We set out to examine the mitochondrial redox responses of a panel of TNBC cell lines of various subtypes to the inhibition of Nampt. We treated TNBC cells with FK866, a specific pharmacological Nampt inhibitor [39,40] that can diminish the NAD pool and cause apoptosis in tumor cells [41]. Figure 1B shows a schematic of our experiments.

**Figure 2:**
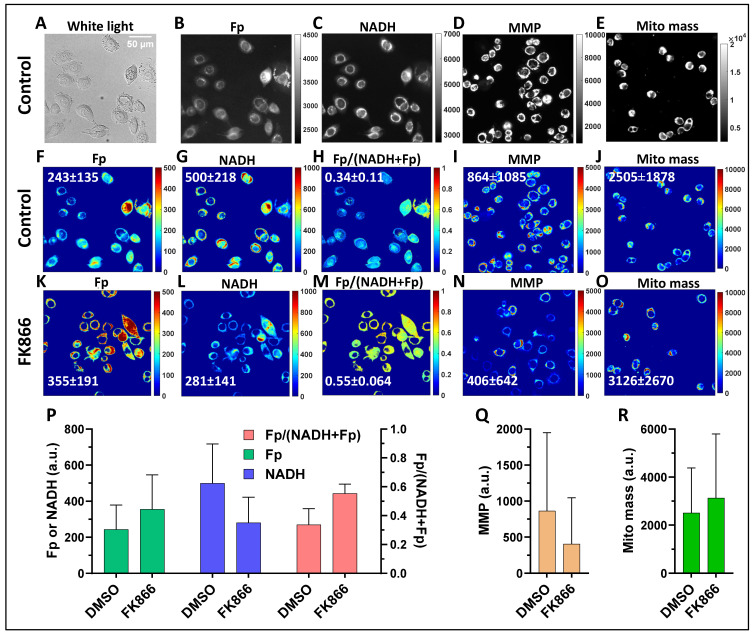
Typical images of MDA-MB-468 cells subjected to various imaging. (**A**–**E**) white light image and raw images of Fp and NADH, mitochondrial membrane potential (MMP) represented by TMRE intensity, and mitochondrial mass represented by MitoView Green intensity, respectively, for control. (**F**–**J**) display the processed images of those shown in (**A**–**E**). (**K**–**O**) the processed images of MDA-MB-468 cells treated with 50 nM FK866 for 48 h. The intensity bars for the raw images are in arbitrary unit and are set for better visualization of signal dynamic range. The intensity bars for the pseudocolored images represent the pixel values of the corresponding images with the redder color indicating higher values. The numbers in white color in the processed images are the means and standard deviations of the respective images. (**P**) the mean values and standard deviations of the redox indices of images shown in (**F**–**H**) (control) and (**K**–**M**) (treated); (**Q**) the mean values and standard deviations of the MMP shown in images (**I**,**N**); (**R**) the mean values and standard deviations of the mitochondrial mass of images shown in (**J**,**O**). The error bars represent the standard deviations of the pixel values in the corresponding images.

Figure 2 displays representative images of MDA-MB-468 cells: white light image (Figure 2A), the raw images of the mitochondrial Fp and NADH (Figure 2B,C), mitochondrial membrane potential (MMP, Figure 2D), mitochondrial mass (Figure 2E) and the corresponding processed pseudocolored fluorescence images of control. The raw images are displayed in arbitrary units to benefit visualization. The nuclei can be observed in both white light and fluorescence images. Most of the fluorescence signals are cytoplasmic, although some are also observed in the nuclei. The pseudocolored fluorescence images provide the net intensities or values of the pixels obtained through background subtraction and signal thresholding. The cell heterogeneity in the respective parameter is more readily observed in the pseudocolored images by various colors. Figure 2K–O are pseudocolored images of the cells exposed to 50 nM FK866 for 48 h. The redox images (i.e., the images of Fp, NADH, Fp/(NADH + Fp)) enable the visualization of metabolic differences between normal and Nampt-inhibited conditions. Compared to the normal condition (Figure 2F–J), the cells exposed to FK866 exhibited higher Fp levels (more red pixels), lower NADH levels (more blue pixels), and higher mitochondrial redox ratios (more yellowish pixels), indicating an oxidized shift of the mitochondrial redox state. Furthermore, FK866 treatment also caused a ~50% drop in mitochondrial membrane potential (MMP) indicated by TMRE intensity (Figure 2N) but the mitochondrial mass (indicated by MitoView green) appeared to be similar to that of the control (Figure 2O). Figure 2P–R delineate the mean values and the spread of the pixel intensities (represented by the standard deviations SD)) of the corresponding parameters in Figure 2F–O.

**Figure 3:**
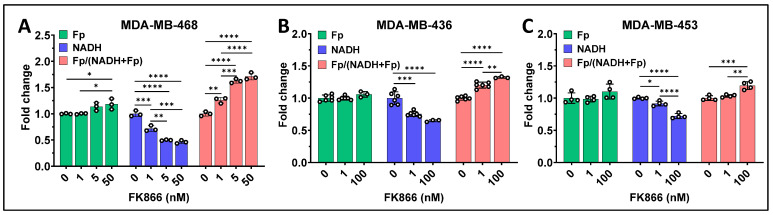
Dose-dependent mitochondrial redox responses observed in the TNBC cells. (**A**) Redox responses of MDA-MB-468 cells; (**B**) Redox responses of MDA-MB-436 cells; (**C**) Redox responses of MDA-MB-453 cells treated with 0–100 nM of FK866 for 48 h. Bars: mean ± SD, black circles indicating individual dishes. *, *p* < 0.05, **, *p* < 0.01, ***, *p* < 0.001, ****, *p* < 0.0001.

We treated the cells with a range of concentrations of FK866 and observed dose-dependent mitochondrial redox changes in MDA-MB-468, MDA-MB-436, and MDA-MB-453 cell lines (Figure 3). Specifically, with increasing FK866 concentration, there were decreasing NADH levels and increasing redox ratios for all three cell lines. Only MDA-MB-468 cells exhibited a significant increase in Fp at 50 nM FK866, whereas an upward trend in Fp was observed at higher FK866 concentration in the other two cell lines. Apparently, compared to the MDA-MB-436 and MDA-MB-453 cell lines treated with 100 nM FK866, the redox state of MDA-MB-468 cells was altered more by FK866 even at half of that concentration.

**Figure 4:**
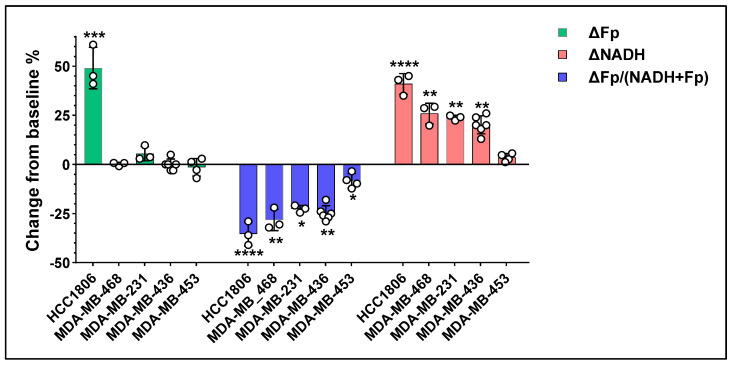
The redox responses represented as the percentage change from baseline for Fp, NADH, and the redox ratio in five human TNBC cell lines treated with 1 nM FK866 for 48 h. Bars: mean ± SD, black circles indicating individual dishes. Asterisks indicate the comparison between the redox indices of control (untreated) and treated cells. *, *p* < 0.05, **, *p* < 0.01, ***, *p* < 0.001, ****, *p* < 0.0001. Note: the data for HCC1806 were extracted from our previous report [35].

**Figure 5:**
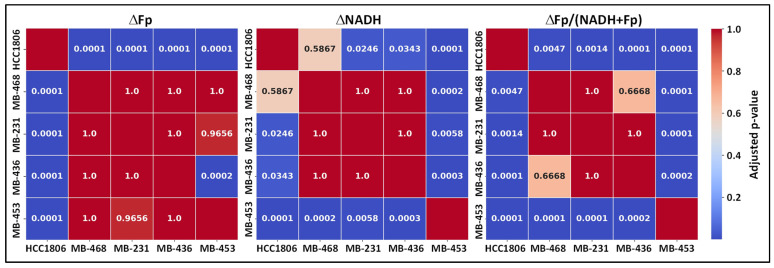
Heatmaps of the adjusted *p* values for multiple comparisons between cell lines of the corresponding redox index changes shown in Figure 4 and Table 2.

Combining our previously published HCC1806 data [35], Table 2 and Figure 4 summarize our ORI findings of these five TNBC lines treated with 1 nM FK866 for 48 h. Overall, compared to the baseline levels (i.e., the redox values of the respective control cells), a significant NADH decrease and a significant increase in the redox ratio after FK866 treatment were observed for all TNBC lines. Among them, only HCC1806 cells exhibited a significant and dramatic Fp increase whereas the other four lines did not. Furthermore, HCC1806 cells showed the greatest change in all three redox indices relative to the other lines tested. Figure 5 summarizes the adjusted *p* values for comparisons of the redox changes between cell lines in heatmaps.

**Figure 6:**
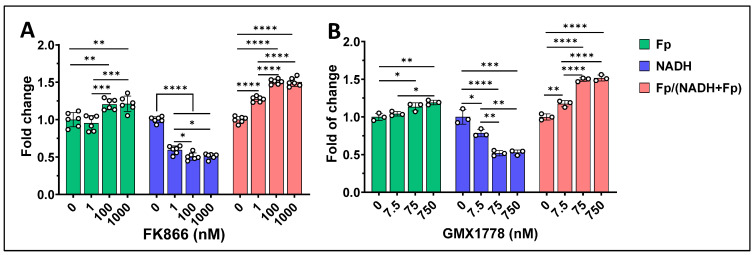
Nampt inhibitor dose-dependent redox changes observed in E0771 cells after 20 h treatment with (**A**) various doses of FK866 and (**B**) various doses of GMX1778. Bars: mean ± SD, black circles indicating individual dishes. *, *p* < 0.05, **, *p* < 0.01, ***, *p* < 0.001, ****, *p* < 0.0001.

We further studied the redox responses of murine TNBC line E0771 treated with FK866 or GMX1778 (another Nampt-specific inhibitor). Figure 6A shows that under 1 nM FK866 treatment for 20 h, NADH significantly decreased by ~35% and the redox ratio increased significantly by ~25%; Fp did not exhibit significant change. Under higher doses of 100 nM and 1000 nM FK866, NADH were further suppressed while Fp and the redox ratio both significantly increased. However, there was no significant difference in any of the redox indices between 100 nM and 1000 nM FK866 treatment, indicating 100 nM FK866 has already induced the largest redox responses in this cell line. GMX1778, in the range of 7.5–750 nM, showed similar effects to FK866 on the redox indices (Figure 6B).

### 3.2. Temporal Changes of the Mitochondrial Redox State Under Nampt Inhibition

**Figure 7:**
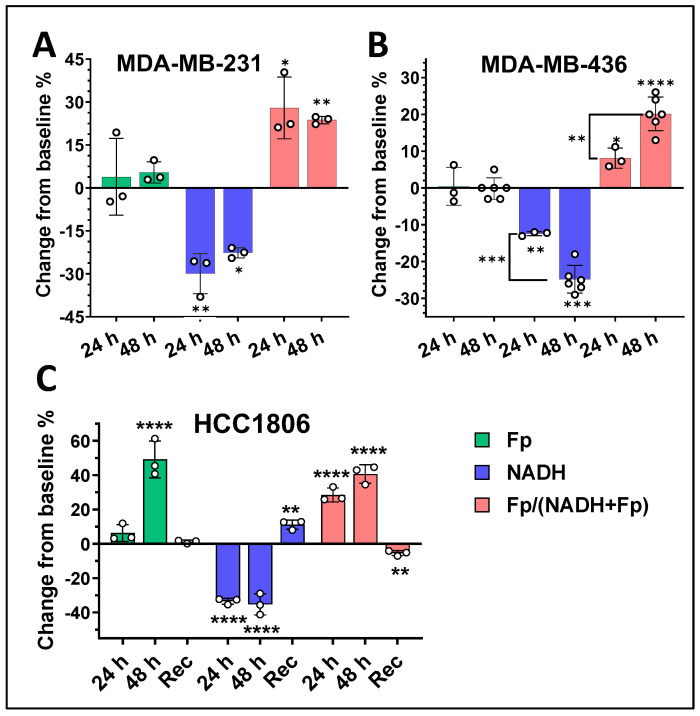
Temporal ORI responses of TNBC cells to FK866 inhibition. (**A**) MDA-MB-231 cells and (**B**) MDA-MB-436 cells to 1 nM FK866 treatment for 24 or 48 h treatment; (**C**) HCC1806 cells were first treated with 1 nM FK866 for 48 h then allowed 24 h to recover (Rec) after removal of FK866. Bars: mean ± SD, black circles indicating individual dishes. Asterisks by themselves indicate the comparisons between the values of control (untreated) and treatment. Asterisks with brackets in (**B**) indicate the comparisons between the values of 24 h and 48 h treatment. *, *p* < 0.05, **, *p* < 0.01, ***, *p* < 0.001, ****, *p* < 0.0001. *p* values for multiple comparisons between different time points in (**B**) were adjusted by Bonferroni post hoc method.

**Figure A2:**
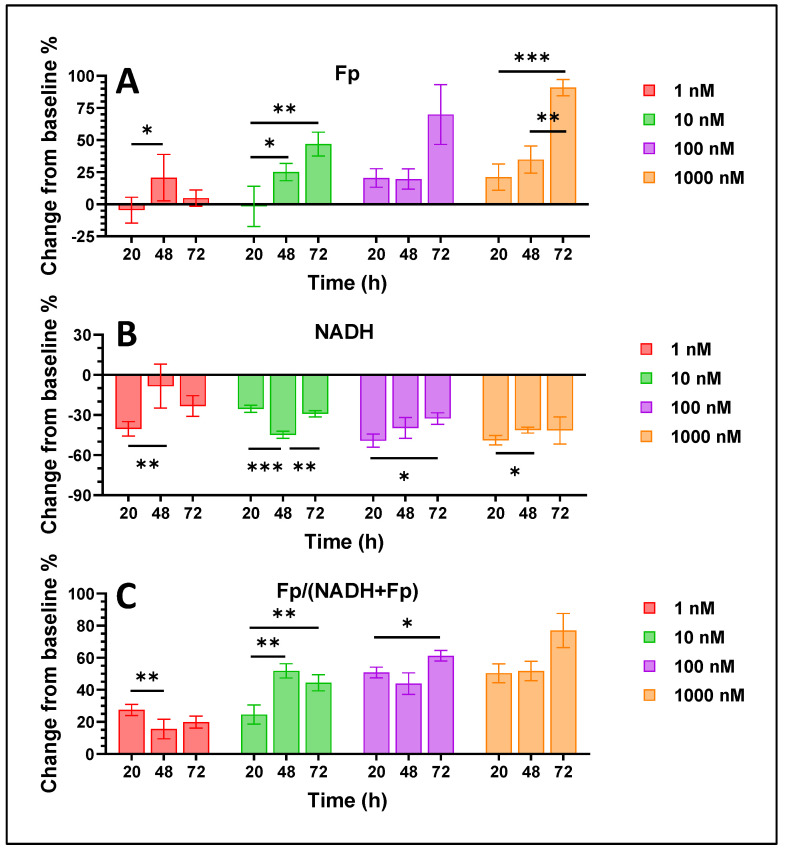
Temporal redox changes of E0771 cells due to various doses of FK866 treatment. Bars: mean ± SD. *, *p* < 0.05, **, *p* < 0.01, ***, *p* < 0.001.

It has been reported that FK866 treatment resulted in a time-dependent change in NAD^+^ level in some breast cancer cell lines [3]. For example, MDA-MB-231 cells displayed a time-dependent decrease in NAD^+^, in which NAD^+^ was the lowest after 24 h but then trended higher after 48 h of treatment, which suggests that NADH may also exhibit similar kinetic behavior. We observed a similar kinetic pattern in NADH (Figure 7A). Interestingly, in MDA-MB-436 cells, we found that NADH was significantly further decreased with additional 24 h treatment and the redox ratio was significantly further increased (Figure 7B). Temporal redox changes of E0771 cells mediated by various doses of FK866 can be found in Figure A2 in Appendix A.

To investigate whether the redox state of TNBC cells may recover after removing FK866, we first treated HCC1806 cells with 1 nM FK866 for 48 h then rinsed the cells with PBS twice. The cells were then incubated in fresh medium without FK866 for 24 h followed by imaging. As shown in Figure 7C, after removal of FK866 and incubation in fresh medium for 24 h, Fp completely recovered to its baseline, while NADH surprisingly elevated ~10% higher than its baseline, resulting in a significant decrease in the redox ratio.

### 3.3. The Effects of NAD^+^ Precursors on the Mitochondrial Redox State

**Figure 8:**
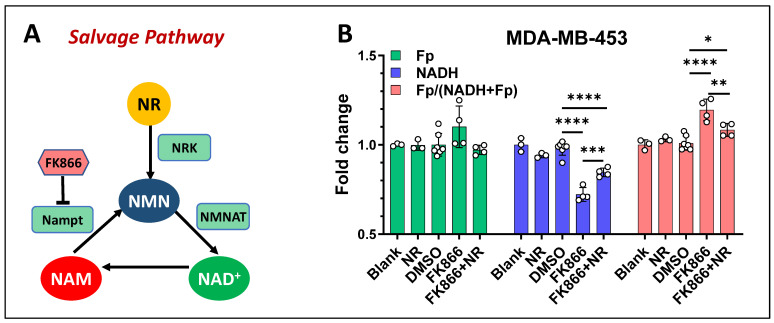
NR rescue effects on MDA-MB-453 cells under 100 nM FK866 for 48 h. (**A**) Both NR and NAM are converted to NMN but via different enzymes. (**B**) NR (1 mM) rescue effect. Bars: mean ± SD, black circles indicating individual dishes. *, *p* < 0.05, **, *p* < 0.01, ***, *p* < 0.001, ****, *p* < 0.0001. Note: all redox indices were normalized to their respective control.

Both nicotinamide riboside (NR) and nicotinamide (NAM) are NAD^+^ precursors but they are converted to NAD^+^ via different pathways (Figure 8A). NR is phosphorylated by Nicotinamide riboside kinase (NRK) to form nicotinamide mononucleotide (NMN), which is then converted to NAD^+^ via nicotinamide mononucleotide adenylyl transferase (NMNAT). These steps to form NAD^+^ are independent of Nampt. As such, when NR is supplemented to cell culture under FK866 treatment, an increase in NADH is expected. We treated MDA-MB-453 cells either with 100 nM FK866 alone, 1 mM NR alone, or co-treated with 100 nM FK866 and 1 mM NR for 48 h. As expected, with NR + FK866 co-treatment, NADH levels were significantly higher than that with FK866 alone, but slightly lower than the untreated control, and the redox ratio was significantly decreased. Fp levels remained unchanged under all treatments (Figure 8B).

**Figure 9:**
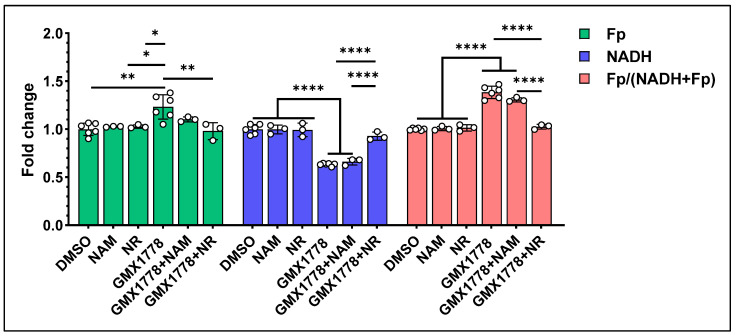
ORI responses of AT-3 cells to Nampt inhibition and NR rescue effect. 500 nM GMX1778, 1 mM NR, or 500 nM GMX1778, 1 mM NR treatment, or NR and NAM co-treatment for 24 h. Bars: mean ± SD, black circles indicating individual dishes. *, *p* < 0.05, **, *p* < 0.01, ****, *p* < 0.0001.

Unlike NR, NAM depends on Nampt to be converted into NMN, which then becomes NAD^+^ (Figure 8A). As such, it is expected that NADH levels of FK866-treated cells will remain low despite NAM supplementation. We treated murine breast cancer cell line, AT-3, either with 500 nM GMX1778 alone, or in combination with NR or NAM. Our findings (Figure 9) showed that GMX1778 significantly lowered NADH and increased Fp and the redox ratio. NR co-treatment significantly raised NADH level whereas NAM co-treatment did not increase NADH. NR co-treatment also significantly lowered Fp level compared to GMX1778 treatment alone.

### 3.4. Correlations Between the Mitochondrial Redox Changes and Mitochondrial Properties and Growth Inhibition

**Figure 10:**
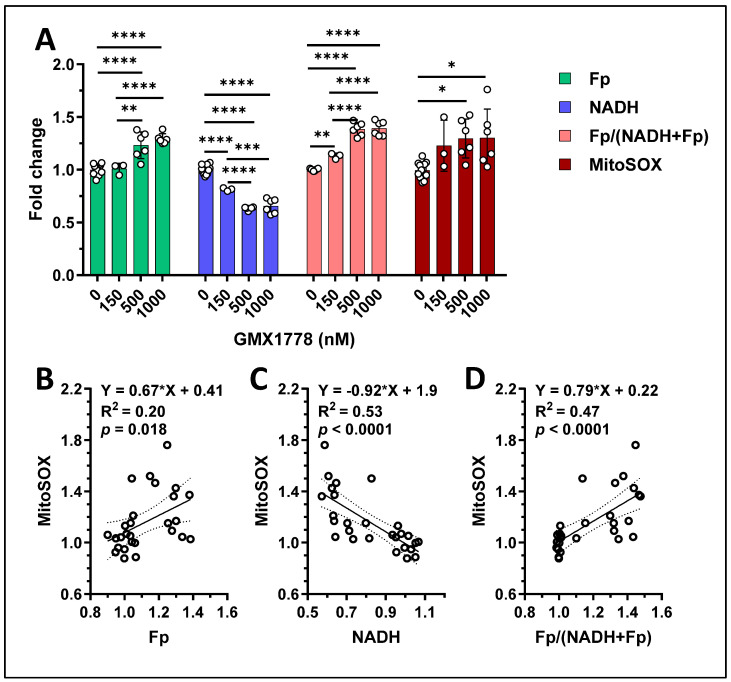
(**A**) ORI responses and changes in mitochondrial ROS of AT-3 cells treated with various doses of GMX1778 for 24 h. (**B**–**D**) The significant linear correlations between mitochondrial ROS and Fp, NADH, and the redox ratio, respectively, determined from data shown in (**A**), where R^2^ and *p* values are indicated on the graphs. Bars: mean ± SD, black circles indicating individual dishes. Dashed lines: 95% confidence intervals *, *p* < 0.05, **, *p* < 0.01, ***, *p* < 0.001, ****, *p* < 0.0001.

One of the advantages of ORI is that it is a label-free technique, allowing other properties to be imaged by adding the relevant fluorescence probes after performing ORI, so that multidimensional information can be obtained from the same cells. Nampt inhibition is known to yield excessive ROS. We investigated the relationship between the mitochondrial redox indices and ROS levels due to Nampt inhibition. AT-3 cells treated with various doses of GMX1778 were first subjected to ORI, followed by incubation with the mitochondrial ROS probe, MitoSOX Red, and then imaged accordingly. Figure 10 displays the quantified redox indices and mitochondrial ROS. With increasing GMX178 dose, NADH decreased, whereas both Fp and the redox ratio increased, and ROS increased in a dose-dependent manner (Figure 10A). Significant linear correlations were found between the mitochondrial redox indices and ROS (Figure 10B–D). Specifically, Fp and the redox ratio positively corelated with ROS and NADH negatively correlated with ROS.

**Figure 11:**
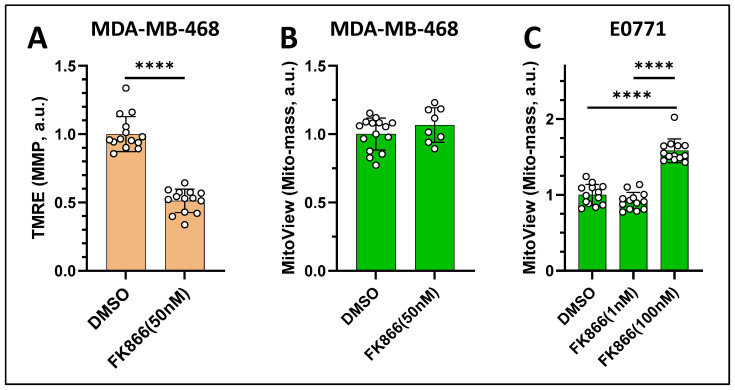
The effects of FK866 on the mitochondrial membrane potential and mitochondrial mass indicated by fluorescence probes TMRE or MitoView Green. (**A**) A large decrease (~40%) in the mitochondrial membrane potential in MDA-MB-468 cells treated with 50 nM FK866 for 48 h; (**B**) insignificant change in the mitochondrial mass of MDA-MB-468; (**C**) dose-dependent increase in the mitochondrial mass of E0771 cells treated with either 1 nM or 100 nM FK866 for 24 h. Bars: mean ± SD, black circles indicating individual FOVs. ****, *p* < 0.0001.

Inhibiting Nampt can also diminish mitochondrial membrane potential and alter mitochondrial mass. Using TMRE as an MMP indicator, we observed ~40% MMP decrease in MDA-MB-468 cells exposed to 50 nM FK866 for 48 h (Figure 11A). On the other hand, MitoView Green as an MMP-independent mitochondrial mass indicator showed insignificant change of the same cells (Figure 11B). In mouse TNBC line E0771, we observed a ~50% increase in mitochondrial mass after 24 h exposure to 100 nM FK866, whereas after exposure to 1 nM FK866 for 24 h, the cells showed no significant mitochondrial expansion (Figure 11C).

**Figure 12:**
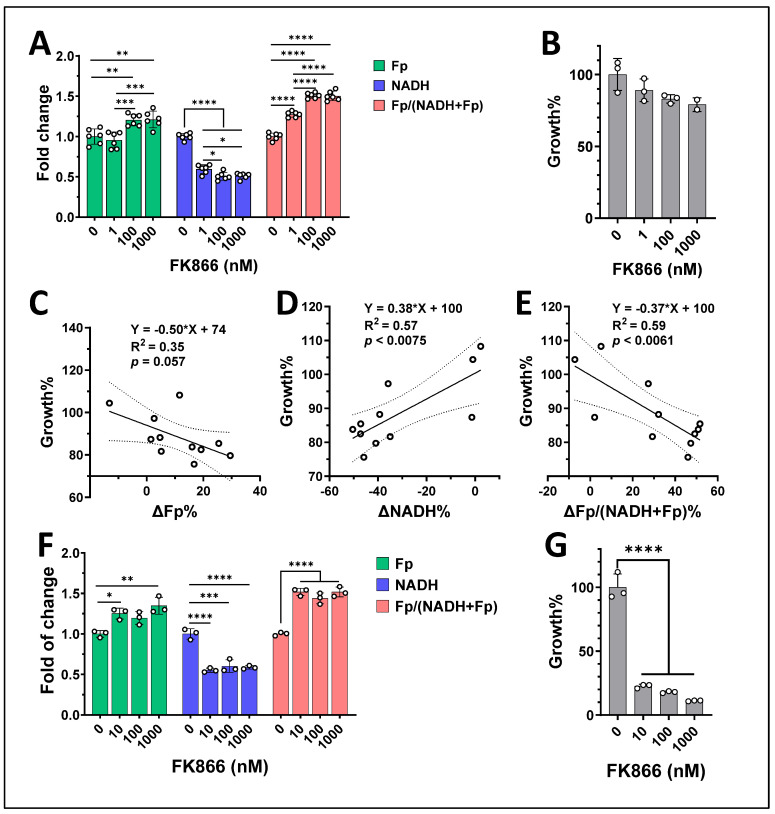
The correlations between the mitochondrial redox indices and cell growth at various doses of FK866 and two treatment periods for E0771 cells. (**A**) The dose-dependent changes of the mitochondrial redox indices (note that this figure is the same as that shown in Figure 6A); (**B**) The dose-dependent cell growth retardation due to 20 h exposure to various doses of FK866; (**C**–**E**) The significant linear correlations between the redox indices and cell growth under 20 h exposure to various doses of FK866, where R^2^ and *p* values are indicated on the graphs. (**F**,**G**) The dose-dependent changes of the mitochondrial redox indices and suppressed cell growth due to 48 h treatment with various doses of FK866, respectively. Bars: mean ± SD, black circles indicating individual dishes. *, *p* < 0.05, **, *p* < 0.01, ***, *p* < 0.001, ****, *p* < 0.0001.

FK866 is known to inhibit cell growth and induce apoptosis in a dose- and time-dependent manner. We next examined whether changes in the mitochondrial redox indices correlated with FK866-induced growth retardation. E0771 cells were treated with various doses of FK866 and for 20 h or 48 h. After performing ORI, we stained the cell nuclei with Hoechst 33342 and imaged the entire dishes to count the total cells in the dishes. With 20 h treatment and increasing FK866 dose, we found that both the increase in Fp (ΔFp) and the increase in the redox ratio (ΔFp/(NADH + Fp)) significantly and negatively correlated with growth, i.e., the larger the increases in Fp and Fp/(NADH + Fp), the stronger inhibition of cell growth, whereas the larger the decrease in NADH (ΔNADH), the stronger the cell growth inhibition was (Figure 12). Furthermore, with prolonged 48 h treatment, there were no further significant changes in the redox indices, whereas cell growth was further suppressed (>50% cells had died).

**Figure 13:**
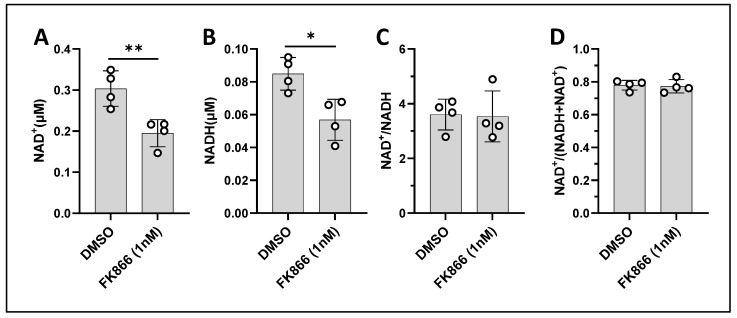
Effects of 1 nM FK866 on NAD^+^ and NADH of E0771 cell homogenates after 24 h exposure. Data were obtained with two technical and two biological replicates. Bars: mean ± SD. *, *p* < 0.05, **, *p* < 0.01.

FK866 is known to diminish the total NAD pool (NAD^+^ and NADH) with varied NAD^+^/NADH ratio depending on the system [42]. We next explored how the FK866-induced mitochondrial redox change was related to the total NAD pool as detected by a commercial NAD biochemical assay kit. Our data showed that 24 h treatment with 1 nM FK866 lowered both NAD^+^ and NADH (thus, the total NAD pool) by ~35% of E0771 cells, whereas the NAD^+^/NADH ratio remained unaffected (Figure 13). On the other hand, our ORI data showed a ~40% decrease in NADH in E0771 cells under the same condition (Figure 6A), largely in agreement with the data obtained from the cell homogenates.

## 4. Discussion

Metabolic phenotyping of TNBC cells under stress enables the identification of druggable targets, responsive subtypes, and treatment response biomarkers, paving the way for the development of combination therapeutic strategies. The present study examined the capability of optical redox imaging to characterize and differentiate among TNBC cells under NAD-deficient metabolic stress caused by pharmacological inhibition of the NAD biosynthesis salvage pathway.

Cancer cells have a high demand for NAD to sustain their rapid growth through enhanced glycolysis and a reductive shift of the NAD^+^/NADH ratio (a more reduced NAD redox state). The NAD^+^ salvage pathway, with Nampt as the rate limiting enzyme, is essential to maintaining the NAD^+^-dependent processes [12,43]. Nampt inhibitors were found to be one of the top candidates to priming and sensitizing TNBC cells to apoptosis [44]. Nampt has also been a considered treatment target of other aggressive cancers [45]. Furthermore, Nampt not only promotes cancer cell proliferation but it can also exit cells to promote an immune suppressive microenvironment via various mechanisms [46,47,48,49], whereas inhibition of NAD metabolism was reported to improve the cytotoxicity of CD8^+^ effector T cells by regulating extracellular adenosine levels in the tumor microenvironment of gastric cancer [50].

TNBC cells vary in Nampt expression and dependence on NAD^+^, which may contribute to varied therapeutic effects [3]. Characterization of metabolic phenotypes of TNBC tumors under NAD-deficient stress may facilitate tailored treatment strategies and better predict prognosis. However, the association between the mitochondrial redox state and the activity of the NAD^+^ salvage pathway in TNBC cells has not been systematically investigated. ORI is a convenient technique that readily provides metabolic information by imaging the fluorescence signals of mitochondrial NADH and Fp and computing their ratio. Nampt-inhibited metabolism displays distinct optical metabolic changes in HCC1806 as shown in our previous work [35]. To test whether ORI can differentiate among TNBC subtypes and select the ones that are more dependent on the NAD^+^ salvage pathway, the current study included additional TNBC lines—three different subtypes of human TNBC and two murine breast cancer lines. We found that while all cell lines tested were sensitive to Nampt inhibition, their mitochondrial redox status exhibited differential changes under the same treatment with 1 nM FK866 (Figure 5 and Figure 7). These results suggest that ORI may potentially provide optical metabolic imaging biomarkers to rapidly differentiate among TNBC tumors and to select metabolically more responsive ones for tailored therapeutic strategies.

Among the five human TNBC lines we studied, HCC1806 cells displayed the greatest response to Nampt inhibition in all three redox indices, MDA-MB-453 displayed the least response, and the other three lines were in between with somewhat similar redox alterations. Notably, under 1 nM Fk866 inhibition for 48 h, only HCC1806 cells had a significant and large increase in Fp, whereas all the other four human TNBC lines showed no significant changes in Fp. Furthermore, despite both HCC1806 and MDA-MB-468 being classified as basal-like subtypes, their mitochondrial redox responses indicated by ΔFp and ΔFp/(NADH + Fp) differed significantly under the metabolic stress of NAD-deficiency, in addition to their differences in the baseline redox status [24]. In HCC1806 cells, neither Fp nor NADH changed with increasing FK866 dosage higher than 1 nM, indicating 1 nM FK866 was sufficient to completely inhibit Nampt [35], whereas, in MDA-MB-468 cells, we saw a significant dose-dependent decrease in NADH and increase in the redox ratio with increasing dose of FK866. On the other hand, both MDA-MB-231 and MDA-MB-436 are MSL subtypes and their redox alterations were similar under 1 nM FK866 despite the differences in their baseline redox indices. However, their temporal redox changes differed (Figure 7).

Among the five human TNBC lines, MDA-MB-453 has the lowest Nampt mRNA expression, sequentially followed by MDA-MB-468, HCC1806, MDA-MB-231, and MDA-MB-436 [3]. The relatively low Nampt mRNA expression of MDA-MB-453 cells potentially explains why the mitochondrial redox status is less sensitive to Nampt inhibition; however, Nampt mRNA expression alone does not seem to explain why the redox status of HCC1806 cells are more sensitive to FK866 than MDA-MB-231 and MDA-MB-436 cells.

Many enzymes are NAD^+^-dependent, notably 3-phosphoglycerate dehydrogenase (PHGDH), the first enzyme of the mammalian serine biosynthesis pathway. Certain breast cancers (especially ER^−^) depend on genomic amplification of PHGDH to divert glycolytic flux into serine and glycine metabolism [51,52] and PHGDH-high breast cancer lines are more susceptible to Nampt inhibition [3]. MDA-MB-436 has both the highest Nampt mRNA expression and PHGDH mRNA expression among the five TNBC lines. As such, one would expect that inhibition of the NAD^+^ biosynthesis salvage pathway should have more significantly affected the NAD levels in MB-436 cells; however, Nampt inhibition did not result in the greatest suppression of NADH level in this cell line. Taken together, neither Nampt mRNA expression nor PHGDG mRNA expression appear to correlate well with the changes in the mitochondrial redox indices, implicating the unique values of ORI for differentiating the metabolic phenotypes of TNBC tumors under the stress of NAD-deficiency and for conveniently selecting the susceptible subtypes.

In our previous work, for cells co-treated with NR and FK866, we observed a NR rescue effect of NADH level in HCC1806 but not in MDA-MB-231, and we observed no Fp change in HCC1806 due to NR [35]. In the present study, we treated AT-3 cells with a combination of either GMX1778 + NAM or GMX1778 + NR. We observed that only NR exhibited a NADH rescue effect and NAM did not, further confirming the specific responsiveness of the ORI technique. Moreover, NR also lowered Fp level and the redox ratio raised by GMX1778 alone.

**Figure A3:**
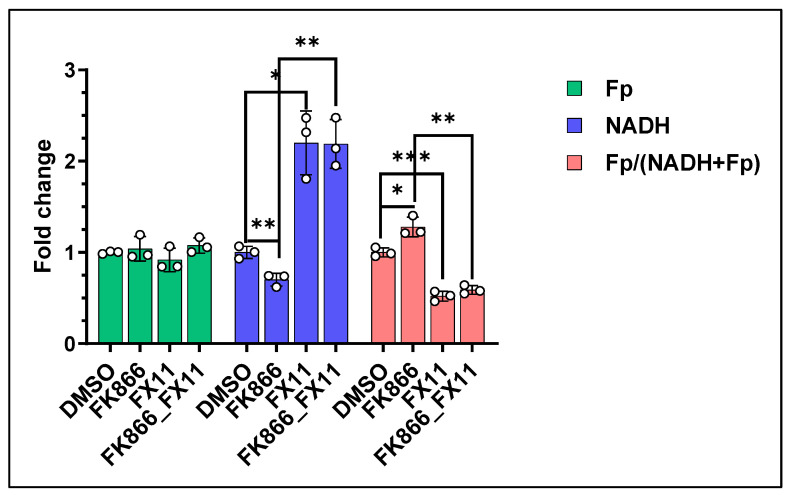
LADH A specific inhibitor FX11 (5 µM) added to MDA-MB-231 cells exposed to 1 nM FK866 for 24 h immediately yielded a large spike of NADH and a reductive shift of the mitochondrial redox state. Bars: mean ± SD, black circles indicating individual samples. *, *p* < 0.05, **, *p* < 0.01, ***, *p* < 0.001.

NAD^+^ is synthesized via three pathways: the de novo pathway through tryptophan, the Preiss–Handler pathway through nicotinic acid (NA), and the salvage pathway through either NAM or NR. Growing evidence suggests that upon Nampt inhibition, other NAD^+^-biosynthesis pathways, such as the Preiss–Handler pathway, can be active and may provide sufficient NAD^+^ in tumor cells [53]. We previously observed a large spike of NADH signal immediately after FX11 (a specific inhibitor of lactate dehydrogenase A) was administered to HCC1806 cells treated with FK866 for 48 h [35]. In this study, we also tested the same concept in MDA-MB-231 cells and observed the similar FX11 effects, i.e., a large spike of NADH in FK866 treated cells (Figure A3). However, whether these results indicate other NAD^+^ biosynthesis pathways were active or NAD was not fully depleted is less clear, although NADH in HCC1806 cells was at the lowest level when we observed the large spike of NADH after FX11. On the other hand, we have previously measured tryptophan incorporation across of a range of cell lines (including MDA-MB-231 and MDA-MB-468 lines) and found that the de novo pathway is not active except in primary hepatocytes [54].

It remains to be investigated whether cells that respond strongly to Nampt inhibition (such as HCC1806 cells) might do so because of a higher NAD^+^ need, or alternatively, a higher dependence on Nampt for their NAD^+^ synthesis. Metabolomic profiling can quantify the intermediates and products which can be tested for their correlations with the ORI readouts. For example, high levels of quinolinic acid suggest an active de novo pathway; accumulation of NA or nicotinic acid mononucleotide indicates the Preiss–Handler pathway; and elevated levels of NMN suggest the salvage pathway, the target of FK866, is active. However, cancer cells are plastic and may prefer certain pathways not only based on their metabolic needs but also the micro-environment, such as pH and pO_2_.

We observed that all three redox indices significantly and linearly correlate with mitochondrial ROS in AT-3 cells induced by various doses ofGMX1778, where lower NADH corresponded to higher ROS; both higher Fp and the redox ratio corresponded to higher ROS. These correlations agree with our previous findings in both cancer and normal cells where exogenous hydrogen peroxide was administered as an acute (3–30 min.) oxidative stressor to test mitochondrial redox responses [35,37,55], supporting the utility of ORI as a label-free technique that serves as a biomarker for both prolonged and acute oxidative stress.

Inhibition of Nampt activity impairs mitochondrial function such as diminishing MMP in many types of cancer cells [50,56,57]. Consistently, we observed a ~50% decrease in MMP in MDA-MB-468 cells exposed to 50 nM FK866, corresponding to diminished NADH and increased mitochondrial redox ratio.

It is known that mitochondrial stress alters cellular metabolism [43]. It was found that TNBC cells underwent mitochondrial rewiring with increased mitochondrial spare respiratory capacity and a higher mitochondrial mass to adapt to FK866-induced NAD(H) shortage [58]. Consistently, we found that E0771 cells adapt to FK866-induced metabolic stress with a higher mitochondrial mass (Figure 11C), indicating that these cells under NAD-deficient stress were compensating their energy needs by increasing the mitochondrial biogenesis.

FK866 inhibits cancer cell growth, ultimately leading to cell death. Our data showed that the changes in NADH and the mitochondrial redox ratio significantly correlated with E0771 cell growth inhibition with 20 h exposure of various doses of FK866, whereas prolonged 48 h exposure yielded significant cell death without further changes in the redox indices. This suggests that mitochondrial redox changes could serve as early biomarkers for treatment response.

Cellular NAD^+^ has much higher concentration than NADH. As FK866 decreases both NAD^+^ and NADH (the total NAD pool) [41], the Fp/NADH ratio can theoretically change due to lower NADH even when NAD^+^/NADH is actually unchanged. For example, in E0771 cells exposed to FK866 for a short time, we observed a ~35% decrease of both NAD^+^ and NADH, whereas the NAD^+^/NADH ratio (and the normalized form NAD^+^/(NADH + NAD^+^)) thus remained unaffected due to the corresponding decreases in both dinucleotides. While the NADH decrease is similar detected with either the NAD biochemical assay or ORI, the mitochondrial redox ratio Fp/(NADH + Fp) had an oxidized shift (~27% higher), which differs from the unaffected NAD^+^/(NADH + NAD^+^) ratio. Importantly, in most cases, a more oxidized shift in the ratio was accompanied by an increase in raw Fp signal, suggesting redox changes even prior to normalizing to NADH (e.g., Figure 12A,F). Furthermore, since the NAD biochemical assay uses cell homogenates, it does not have the power to resolve the compartmental/subcellular NADH content. On the other hand, the optical redox imaging technique provides the compartmental/subcellular NADH and the signals are more specific to mitochondria where the bound form of NADH are more abundant and bound NADH has 10 times higher quantum yield than the free form. From the perspective of a biomarker, the ability of the signal to differentiate cell behavior is critical regardless of whether it primarily measures changes in redox or total NAD^+^; and NADH.

ORI involves two redox sensors, representing the redox status of two redox pairs, i.e., the NADH/NAD^+^ and FAD/FADH_2_ pairs. The redox ratio, Fp/NADH (or Fp/(NADH + Fp) in normalized form), reflects the balance of these two redox pairs. It was shown that there is a linear correlation between acetoacetate/3-hydroxybutyrate in arterial blood and Fp/NADH in freeze-trapped human liver tissue [59]. Furthermore, a positive linear correlation between LC/MS determined NAD^+^/(NADH + NAD^+^) and ORI-determined Fp/(NADH + Fp) was demonstrated in various types of engineered epithelial tissue equivalents [60]. However, evidence also suggests that there might be some cases where these two redox ratios do not always positively correlate. For example, in aging tissues, there is a reductive shift of NAD^+^/NADH ratio, whereas Fp/NADH shifts to a more oxidized ratio [61,62,63] and what we found in E0771 cells treated with FK866 in the present study. As such, it would be interesting and relevant to learn whether changing NAD(H) concentration might shift the Fp/NADH ratio more than the NAD^+^/NADH ratio with Nampt inhibition in different TNBC subtypes.

Previously, we characterized the basal mitochondrial redox state of four of the five human TNBC lines included in this study and found that the basal redox ratio differs among the subtypes and there was a positive correlation between the redox ratio and the invasive potential [24]. Furthermore, TGF-β/TGF-βR signaling is known to promote cancer cell migration by inducing epithelial–mesenchymal–transition (EMT). We showed that the basal mitochondrial redox state had a reductive shift in response to TGF-β/TGF-βR signaling and corresponded with increased migration capacity in both HCC1806 and MDA-MB-231 [64]. These data suggest involvement of the mitochondrial redox status in breast cancer invasiveness. The results reported in the present study further demonstrated the utility of ORI for metabolic phenotyping of TNBC cells under NAD-deficient stress. Future work can examine how invasiveness is affected by Nampt inhibition in these TNBC lines and test whether the altered redox ratio still correlate with invasiveness of TNBC cells under NAD-deficiency.

Future in-depth investigation can also be carried out to assess the effects of genetic modifications of the NAMPT expression to determine whether the loss of NAMPT function elicits redox changes similar to those observed with pharmacological inhibition in TNBC cell lines. While Nampt down-regulation and FK866 may have comparable impacts on the redox status through NAD^+^; depletion, FK866 is a more controlled and tunable experimental tool. However, genetic loss of function may better reflect chronic effects and compensatory mechanisms.

ORI as a label-free technique has certain advantages in live cell imaging, especially in in vitro metabolic studies, where the same samples can generate multidimensional data by adding the relevant fluorescence probes after ORI. ORI has been utilized to detect the therapeutic responses of live cells as demonstrated by our works [35,64,65] and the works of many other researchers [19,20,21,22,66,67,68], displaying shifted mitochondrial redox states due to pharmacological or metabolic stress. In addition to detecting treatment responses, ORI is sensitive to cellular oxidative stress. These findings all support the utility of ORI in detecting treatment response and metabolic phenotyping of TNBC cells, thus potentially guiding the therapeutic strategies.

Despite the many advantages, ORI has limitations, including its relatively lower and confounded endogenous fluorescence signals. For example, since the fluorescence spectra of NADH and NADPH are identical, ORI does not differentiate their signals and the reported NADH values are co-contributed by both despite that the NADPH contribution is usually much smaller.

## 5. Conclusions

This study investigated the utility of the optical redox imaging technique in differentiation among the subtypes of TNBC cells under the metabolic stress induced by inhibited NAD^+^ biosynthesis of the salvage pathway. Our results showed that while diverse subtypes of TNBC were sensitive to Nampt inhibition in a dose- and time-dependent manner, they had differential responses in the mitochondrial redox state. We also showed significant correlations between the mitochondrial redox indices and ROS as well as growth inhibition. Following ORI, we explored the alterations in other mitochondrial properties caused by Nampt inhibition through exogenous fluorescence probes. Our data support the utility of ORI in phenotyping of TNBC cells under NAD-deficient metabolic stress to facilitate combination therapy strategies and identify potential biomarkers of treatment response.

## Figures and Tables

**Table 1:** The microscope settings for all imaging sessions.

Channel	Excitation (nm)	Emission (nm)	LED Power	Exposure (s)	Gain	Incubation (min)
NADH	370–400	414–450	25%	2	1	
Fp	450–488	500–530	40%	3	1	
MitoView Green	450–488	500–530	20%	0.3	1	20
TMRE	540–570	580–610	40%	0.05	1	20
Hoechst 33342	370–400	414–450	25%	0.15	1	10
MitoSOX Red	370–400	580–610	40%	0.2	1	10

**Table 2:** The changes in the redox indices due to 48 h of 1 nM FK866 treatment.

Cell Line	ΔFp%	ΔNADH%	Δ(Fp/(NADH + Fp))%
HCC1806	49 ± 11	−35 ± 6.2	41 ± 5.4
MDA-MB-468	0.16 ± 0.93	−28.2 ± 5.4	25.9 ± 5.3
MDA-MB-231	5.4 ± 3.7	−22.6 ± 1.8	23.7 ± 1.3
MDA-MB-436	0.13 ± 3.0	−24.54 ± 3.8	20.21 ± 4.6
MDA-MB-453	1.4 ± 4.47	−8.4 ± 3.7	3.5 ± 2.1

**Figure A1:**
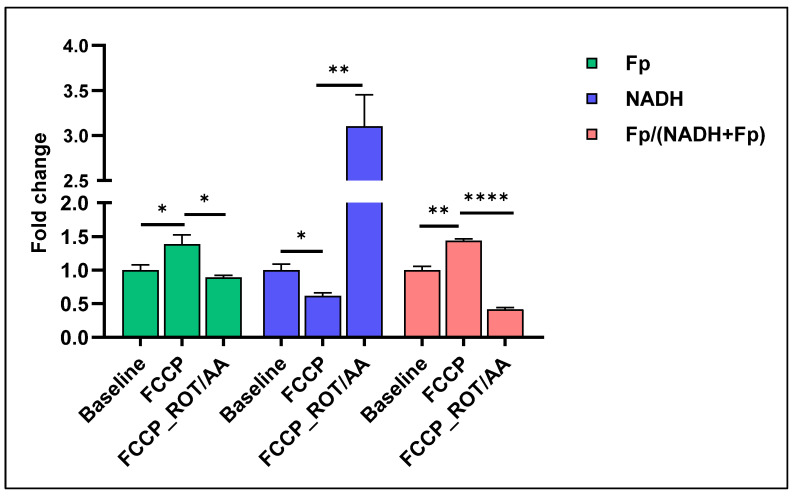
To confirm the redox responses to metabolic modulations, we performed redox titration using MDA-MB-468 cells under normal growth conditions. We observed the expected changes, i.e., a decrease in NADH due to uncoupled oxidative phosphorylation from the electron transport chain by mitochondrial uncoupler FCCP, followed by an increase in NADH due to the inhibition of complex I and III by rotenone and antimycin A (ROT/AA), respectively. Bars: mean ± SD, N = 3–4 FOVs. *, *p* < 0.05, **, *p* < 0.01, ****, *p* < 0.0001.

## Data Availability

The relevant data will be provided upon reasonable request.
